# Identifying Electrophysiological Prodromes of Post-traumatic Stress Disorder: Results from a Pilot Study

**DOI:** 10.3389/fpsyt.2017.00071

**Published:** 2017-05-15

**Authors:** Chao Wang, Michelle E. Costanzo, Paul E. Rapp, David Darmon, Kylee Bashirelahi, Dominic E. Nathan, Christopher J. Cellucci, Michael J. Roy, David O. Keyser

**Affiliations:** ^1^Traumatic Injury Research Program, Department of Military and Emergency Medicine, Uniformed Services University of the Health Sciences, Bethesda, MD, USA; ^2^The Henry M. Jackson Foundation for the Advancement of Military Medicine, Inc., Bethesda, MD, USA; ^3^Department of Medicine and Center for Neuroscience and Regenerative Medicine, Uniformed Services University of the Health Sciences, Bethesda, MD, USA; ^4^Graduate School of Nursing, Uniformed Services University of the Health Sciences, Bethesda, MD, USA; ^5^Aquinas LLC, Berwyn, PA, USA

**Keywords:** post-traumatic stress disorder, prodromes, event-related potentials, delayed onset, traumatic brain injury, P300

## Abstract

The objective of this research project is the identification of a physiological prodrome of post-traumatic stress disorder (PTSD) that has a reliability that could justify preemptive treatment in the sub-syndromal state. Because abnormalities in event-related potentials (ERPs) have been observed in fully expressed PTSD, the possible utility of abnormal ERPs in predicting delayed-onset PTSD was investigated. ERPs were recorded from military service members recently returned from Iraq or Afghanistan who did not meet PTSD diagnostic criteria at the time of ERP acquisition. Participants (*n* = 65) were followed for up to 1 year, and 7.7% of the cohorts (*n* = 5) were PTSD-positive at follow-up. The initial analysis of the receiver operating characteristic (ROC) curve constructed using ERP metrics was encouraging. The average amplitude to target stimuli gave an area under the ROC curve of greater than 0.8. Classification based on the Youden index, which is determined from the ROC, gave positive results. Using average target amplitude at electrode Cz yielded Sensitivity = 0.80 and Specificity = 0.87. A more systematic statistical analysis of the ERP data indicated that the ROC results may simply represent a fortuitous consequence of small sample size. Predicted error rates based on the distribution of target ERP amplitudes approached those of random classification. A leave-one-out cross validation using a Gaussian likelihood classifier with Bayesian priors gave lower values of sensitivity and specificity. In contrast with the ROC results, the leave-one-out classification at Cz gave Sensitivity = 0.65 and Specificity = 0.60. A bootstrap calculation, again using the Gaussian likelihood classifier at Cz, gave Sensitivity = 0.59 and Specificity = 0.68. Two provisional conclusions can be offered. First, the results can only be considered preliminary due to the small sample size, and a much larger study will be required to assess definitively the utility of ERP prodromes of PTSD. Second, it may be necessary to combine ERPs with other biomarkers in a multivariate metric to produce a prodrome that can justify preemptive treatment.

## Introduction

Historically, psychiatric practice has been reactive rather than preemptive. It has been recognized that a transition to preemptive psychiatry requires the identification of prodromes of psychiatric disorders that have a predictive reliability that justifies intervention in the absence of a fully expressed disorder. A prodrome is not a risk factor. A prodrome is a physiological change antecedent to a full expression of the disorder. Costello and Angold (1) provide the following definition: “… a prodrome is a premonitory manifestation of the disease. It is not a characteristic of the individual or their environment or a causal agent of the disease. A prodromal symptom may or may not continue to be manifest once the full disease appears. Conversely, the same disease may or may not manifest prodromal symptoms in different episodes.” Emerging genetic, epigenetic, and psychophysiological technologies offer the possibility of identifying prodromes or combinations of prodromes (where a combination of metrics may improve specificity) that can warrant preemptive treatment (2, 3). Prior research has investigated prodromes of several psychiatric disorders including psychosis (4–7), depression (8), autism (9, 10), dementia (11), alcoholism and substance abuse (1, 12), and post-traumatic stress disorder [PTSD (13–15)].

The objective of this research project is the identification of a physiological prodrome of PTSD that has a reliability that could justify preemptive treatment in the sub-syndromal state. The search for statistically reliable prodromes requires two things: a sub-syndromal period where physiological changes prior to the disease onset have been initiated, and a measure that can quantify these changes. In the ideal case, a third element can facilitate the search for prodromes: the identification of an at-risk population because an enriched population (a population where incidence is higher than the general population) will increase the statistical likelihood of identifying a prodrome. In this contribution, we address a specific question: can event-related potentials identify individuals at risk of delayed-onset PTSD? As preceding questions we must ask whether an at-risk population can be identified and if there is evidence indicating that PTSD can, in some instances, present with delayed onset? It is the period between trauma exposure and the presentation of a fully expressed PTSD that provides the window of opportunity for preemptive treatment.

### Can a PTSD At-Risk Group Be Identified?

Military deployment is a risk factor for PTSD. The reported incidence of PTSD in veterans varies greatly between studies. A critical review found that PTSD incidence in US Iraq veterans ranges from 4 to 17% (16). Reports of the incidence of PTSD in the general population are similarly varied, but the National Comorbidity Survey Replication Study (17, 18) estimated the lifetime prevalence of PTSD in adult Americans to be 6.8%. Current past year prevalence was estimated at 3.5%. This suggests that military service members (SMs) who have returned from deployment will provide a statistically enriched population increasing the likelihood of identifying prodromes of PTSD. When making this observation, it is recognized that it is possible that military-related PTSD and PTSD in civilian populations may have distinct pathophysiological etiologies. This would potentially limit the general utility of results obtained with a military population.

### Can PTSD Present with Delayed Onset?

Meta-analysis indicates that approximately 25% of PTSD cases present with delayed onset, where delayed onset is defined as meeting diagnostic criteria after a sub-syndromal or asymptomatic period of at least 6 months after the precipitating traumatic event (19, 20). In a military population, Grieger et al. (21) found that the majority of individuals PTSD-positive 7 months after serious combat injury did not meet diagnostic threshold at 1 month post-injury. In cases of PTSD following mild traumatic brain injury (TBI), the fraction of cases presenting with delayed onset can be higher. Bryant et al. (22) found that of those who met PTSD criteria at 24 months following a TBI, 44.1% reported no PTSD at 3 months. The analysis of Smid et al. (20) and Andrews et al. (19) indicates that PTSD can present after a symptom-free period, but it has been found to be more likely after a period of sub-syndromal PTSD in which two or three of the symptom clusters are endorsed (22). The factors contributing to delayed-onset PTSD in the absence of mild TBI are incompletely understood (15). On reviewing the trajectories of full and sub-syndromal PTSD, Bryant et al. (22) reached the following conclusions: “The present study demonstrates longitudinally that there is not a linear relationship between acute trauma response and long-term PTSD and highlights that PTSD levels fluctuate markedly in the initial years after trauma exposure. This pattern can explain the modest predictive capacity of acute markers to identify subsequent PTSD status. The complexity of these trajectories is further indicated by the delayed occurrence of PTSD responses, which appears to result from a combination of the immediate stress response and cumulative stress in the aftermath of the trauma.” These clinical observations further encourage the search for reliable physiological prodromes of PTSD.

### Is There a Prior Literature Reporting Alterations of Event-Related Potentials in Fully Expressed PTSD?

As noted above, an additional requirement in the search for prodromes is the identification of a measure that can quantify physiological changes antecedent to disease onset. This search can be informed by asking whether there are markers that show alteration in the fully expressed disease, since it seems possible that these alterations may have begun prior to reaching diagnostic threshold. In the specific context of this investigation, this question becomes is there a prior literature showing abnormalities in event-related potentials in PTSD patients? An examination of the prior literature summarized in Table 1 suggests that event-related potentials can be altered in the fully expressed PTSD state.

**Table 1:** **Studies reporting ERP abnormalities in PTSD-positive participants**.

Study	Reported observation(s)
Araki et al. (23)	Lower amplitude ERPs at Pz in an auditory oddball task
Blomhoff et al. (24)	Amplitudes to emotionally related words were significantly related to CAPS scores
Charles et al. (25)	P300 amplitude lower in PTSD-positive participants
Felmingham et al. (26)	Auditory oddball, PTSD positive participants show the following
	Target stimuli: reduced P200 amplitude, reduced P300 amplitude, increased N200 amplitude, increased N200 latency, increased P300 latency
	Standard stimuli: reduced P200 amplitude
Ghisolfi et al. (27)	PTSD positive participants showed auditory P50 sensory gating deficits
Hansenne (28)	Literature review includes PTSD
Javanbakht et al. (29)	Literature review of 36 studies. Increased P300 response to trauma-related stimuli. P50 studies suggest impaired gating
Johnson et al. (30)	P300a, P300b amplitudes larger with trauma related stimuli
	P300b small with neutral stimuli
	P300 working memory amplitudes smaller
Karl et al. (31)	Reduced P50 suppression
	Increase P300 amplitude to trauma-related stimuli
Kimble et al. (32)	Significant P300 amplitude enhancements to distracting stimuli
Kimble et al. (33)	Larger frontal, smaller central, and parietal CNVs
McFarlane et al. (34)	Delayed N200 P300 elicited by target and distracter tones indistinguishable
Metzger et al. (35)	Parietal P300 amplitude to target tones were smaller in unmedicated PTSD positive participants
Metzger et al. (36)	Modified Stroop task for personal traumatic, personal positive, and neutral words. PTSD-positive participants have reduced and delayed P300 across word type
Metzger et al. (37)	Contrary to previous results, the PTSD group had larger P300b amplitude and increased P200 amplitude/intensity slopes
Neylan et al. (38)	Impaired P50 gating to non-startle trauma-neutral auditory stimuli
Neylan et al. (39)	Nine of 24 P300 measures were significantly less predictable over time in the PTSD-positive group
Shu et al. (40)	mTBI only compared against mTBI + PTSD, larger emotional face processing ERPs in mTBI + PTSD
Shu et al. (41)	mTBI only compared against mTBI + PTSD, larger inhibitory processing ERPs in mTBI + PTSD
Shucard et al. (42)	PTSD group has longer P300 latency to NoGo stimuli and greater P300 amplitude to irrelevant non-target stimuli

*CAPS, Clinician-Administered PTSD Scale; CNV, contingent negative variation; ERP, event-related potential*.

The divergence of electrophysiological results across studies is consistent with the emerging understanding that PTSD is not a discrete clinical entity and that different pathophysiological processes may be active in different individuals. The results do, however, suggest that alterations of brain electrical behavior can be associated with the disorder. As indicated in Table 1, alterations in P300 are most frequently reported.

There is an emerging understanding of the neurological origin of the empirical results reported in Table 1 that suggests why alterations of P300 may be associated with both fully expressed PTSD and the sub-syndromal state. P300 has been hypothesized to reflect neural activity associated with attention and subsequent memory processing (43), with larger P300 amplitude associated with greater attentional resources employed in the task (44, 45). The prior studies with PTSD positive participants reporting reduced P300 amplitude to target stimuli in the PTSD group compared to the control group, suggest impairment of attentional processes which is consistent with clinical observation. In addition, a meta-analysis examining ERP components and PTSD revealed that the P300 amplitude may also be sensitive to contextual cues such that information processing is modulated based on the situation and environment (31). These dynamics are consistent with functional changes of two reported neural generators of the P300 (46, 47): the anterior cingulate cortex (ACC) and the hippocampus, which are also altered in individuals with PTSD (48). The ACC is critical to attentional processing and fear inhibition (49, 50) and the hippocampus is involved in memory and contextual representations (51). Araki et al. (23) revealed that lower P300 amplitude in patients with PTSD was associated with smaller ACC volume, which linked the P300 abnormality to underlying brain morphological abnormality.

It should be recognized that the results in Table 1 were obtained from participants who were diagnostically PTSD-positive at the time of recording. The question of the utility of ERPs as a predictor of a transition to PTSD is not addressed by these studies, but these studies do suggest that altered ERPs may be present in the sub-syndromal state. This possibility is investigated in this study. The study was sponsored by the Department of Defense to investigate the utility of using a reduced montage that could be implemented in a military field hospital environment. Event-related potentials can be elicited by visual, auditory, somatosensory, and olfactory stimuli, with visual and auditory stimuli being the most commonly used. Hearing and vision can be compromised after blast exposure, but visual disturbances typically resolve faster. We therefore used visual stimuli in this study. As indicated in Table 1, several ERP components [P50, P200, N200, and contingent negative variation (CNV)] can be altered in PTSD-positive participants. Typically, however, the P300 is the most robust component. Since the object of this research program is the development of a robust technology that can be implemented in an austere medical environment, we focused on the P300.

## Methods

### Subjects

We recruited 85 military SMs within 2 months of their return from an Operation Enduring Freedom (OEF)/Operation Iraqi Freedom (OIF) deployment of at least 3 months’ duration in either Iraq or Afghanistan. The Clinician-Administered PTSD Scale (CAPS) (52) and the PTSD Checklist-Military Version (PCL-M) (53) were administrated to assess PTSD. Patient Health Questionnaire-9 (PHQ-9) (54) and the International Classification of Diseases, 10th Clinical Modification (ICD-10) criteria for postconcussional syndrome (PCS) were administrated to determine the presence of depression and PCS, respectively. Exclusion criteria included a history of head injury resulting in loss of consciousness for 60 min or more; a current Glasgow Coma Scale less than 13; visual acuity lower than 20/100 after correction; psychosis; active suicidal, or homicidal ideation; pregnancy; a diagnosis of PCS according ICD-10, PHQ-9 score greater than or equal to 10; and a PCL-M score greater than or equal to 50, or a diagnosis of PTSD made by an experienced psychologist using the CAPS based on the DSM-IV criteria. All subjects provided written informed consent in accordance with the protocol approved by institutional review boards at Uniformed Services University, Walter Reed National Military Medical Center, and the National Institutes of Health.

Out of the 85 participants, 8 were excluded after baseline assessment: 2 for PCL-M ≥50, 2 for PHQ-9 scores ≥10, and 4 for problems with electroencephalogram (EEG) recording. Among the remaining 77 participants, 65 completed at least one follow-up psychological evaluation (52 at 3 months, 33 at 6 months, and 53 at 12 months). On serial follow-up evaluations, 5 of the 65 participants developed PTSD as determined by PCL-M scores (4 PTSD, 1 PTSD with depression). We therefore separated the 65 participants into 5 cases (referred to as Converters, mean age 35.6 ± 6.2 years, 4 men and 1 woman) and 60 controls (referred to as Stables, mean age 30.5 ± 8.0 years, 54 men and 6 women). The 5 Converters and 60 Stables are the final set of subjects in this study. In this paper, we focus on electrophysiological data from baseline assessment as we are trying to identify neural markers that predict the development of PTSD.

All participants in the group of 65 were exposed to relatively severe traumatic experiences. The types of index trauma reported by those who developed PTSD included experiencing a base attack (e.g., mortar or rocket fire, *n* = 1), engaging in combat-related violence (e.g., firefights, hit by improvised explosive device, IED, killing enemy, *n* = 2), witnessing combat-related violence (e.g., watching truck in convoy hit by an IED, witnessing death *n* = 1), and deployment bullying and abuse (*n* = 1). Those who did not develop PTSD also reported experiencing base attacks (*n* = 24), engaging in combat-related violence (*n* = 23), and witnessing combat-related violence (*n* = 13). Two factors, however, preclude a meaningful search for correlations between ERP abnormalities and cause of trauma. The first is the small size of the study population. The second would be applicable even in a larger study. Many, if not most of these participants have received multiple traumas from many causes.

### Electrophysiological Recording

A visual oddball task was performed by subjects in an acoustically and electrically shielded room. Visual stimuli were presented by a digital tachistoscope of our own design and construction. The tachistoscope is a 5 × 5 square array of yellow, light-emitting diodes. Each diode is 1 cm in diameter. Given spacing between LEDs, the array is 6 cm × 6 cm. The standard visual stimulus was a vertical stimulus which consists of the five vertical center line LEDs illuminated simultaneously for 40 ms. The target visual stimulus was a horizontal stimulus which is composed of the five horizontal center line LEDs illuminated simultaneously for 40 ms. Each subject received 125 stimuli in total, of which about 21% (26 ± 1 trials) were target and 79% (99 ± 1 trials) were standard stimuli. The subjects were instructed to maintain a silent count of the number of target stimulus presentations and to report their count at the end. The inter-stimulus onset time was varied randomly between 1.4 and 1.8 s. The number of trials in the current study is sufficient to elicit a valid P300 response. For example, a classic P300 study by Pollich et al. (55) used 25 target trials. Cohen and Polich (56) found that the P300 stabilized with approximately 20 trials.

The scalp EEG was recorded using the EPA6 amplifier (Sensorium Inc.) and the Grass electrodes (Natus Neurology Inc.) at Fz, Cz, Pz, Oz, C3, and C4 according to the standard 10-20 electrode system, with linked earlobes as reference and a forehead ground. Electrode impedances were maintained under 5 kΩ. EOG was recorded from two electrodes placed below and above the right eye. The sampling rate was 2,048 Hz, and the analog filter band-pass was 0.02–500 Hz.

### Data Processing of Electrophysiological Data

Data processing was performed offline using custom scripts written in MATLAB (www.mathworks.com). Channels contaminated by artifacts were removed from analysis. This resulted in one Fz channel (from the Stable group) and four Oz channels (one from the Converter group and three from the Stable group) being removed. EOG artifacts were corrected by using a regression approach (57). The data after EOG correction were high-pass filtered at 0.5 Hz, low-pass filtered at 50 Hz, and down sampled to 256 Hz. The analysis period was −200 to 1,000 ms where time zero denotes stimulus onset. Trials with peak potentials exceeding 75 μV or exhibiting abnormal trends were excluded from ERP averaging. The overall trial rejection rate was 4.84%. Target trials and standard trials were averaged separately. P300 amplitude was measured as the voltage of the largest positive peak of target ERP within 250–500 ms. P300 latency was measured as the time from stimulus onset to the maximum positive amplitude within 250–500 ms.

### Statistical Analyses

Differences between groups in demographics, psychological measures, and task performance (accuracy of target count) were examined by Student’s *t*-tests if data are numerical or Fisher’s exact tests if data are categorical. Because the Oz channel was lost in some recordings (including one in the Converter group), the statistical analysis is limited to Fz, Cz, Pz, C3, and C4 electrode sites. Group differences in P300 amplitude and latency at each electrode site were tested by Student’s *t*-tests. Correlations between P300 amplitude and the psychological measures were examined by Pearson’s correlation coefficient. *p*-Values less than 0.05 were considered statistically significant.

To examine the efficacy of using P300 amplitude as the predictor for PTSD, we performed several statistical analyses including approximate classification error rate, receiver operating characteristic (ROC) curve, leave-one-out cross validation, and bootstrapping. The detailed mathematical methods and equations can be found in the Mathematical Appendices.

## Results

### Subject Characteristics and Baseline Psychological Measures

The subject characteristics and baseline psychological measures were summarized in Table 2. Age, gender, handedness, and history of mild TBI (mTBI) were not significantly different between the Converter and Stable groups. At the baseline assessment, the Converter group reported significantly higher CAPS, PHQ-9, and PCL-M scores than the Stable group.

**Table 2:** **Subject characteristics and baseline psychological measures**.

Variable	Converter (*n = 5*)		Stable (*n = 60*)		Group comparison^a^		
	Mean	SD	Mean	SD	df	*t*-Value	*p*-Value
Age	35.6	6.2	30.5	8.0	63	1.37	0.18
Gender, male/female	4/1		54/6				0.36
Handedness, R/L	5/0		55/5				0.66
History of mTBI < 10 years, yes/no	2/3		18/42				0.33
Clinician-Administered PTSD Scale total	30.6	15.4	18.7	12.5	63	2.02	0.047
Patient Health Questionnaire-9 score	5.2	2.3	2.5	2.3	62	2.51	0.015
PTSD Checklist-Military Version (PCL-M) score	33.4	11.0	25.9	7.4	63	2.10	0.040

*^a^Fisher’s exact tests were used for gender, handedness, and history of mTBI. Student’s *t*-tests were used for other variables*.

### Behavioral Data

The accuracy of target count at baseline assessment was not significantly different between Converters and Stables. For Converters, the mean accuracy of target count was 93.1% (SD 5.0%) and for Stables the mean accuracy was 97.4% (SD 5.5%) The difference was not statistically significant (*t* = 1.70, df = 63, *p* = 0.095).

### P300 Data: Amplitude and Latencies of Averaged Responses

We computed the approximate signal-to-noise ratios (SNRs) for both target and standard trials within the P300 time window for each subject. The SNR was calculated from the power of the ERP during the P300 window (300–400 ms) minus the power of the ERP during baseline (−200 to 0 ms) and then divided by the power of the ERP during baseline window. The mean SNR for single subject ERP for target trials at Pz is 145 (21.6 dB). The mean SNR for single subject ERP for standard trials at Pz is 87 (19.4 dB).

**Figure 1:**
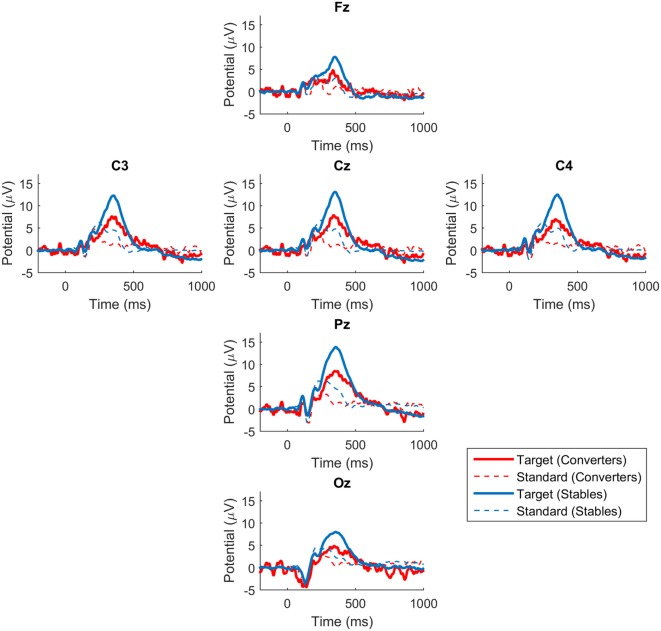
**P300 waveforms in converters and stables**. Grand average ERPs in response to target and standard stimuli at the six electrodes. Blue lines represent waveforms for Stables. Red lines represent waveforms for Converters.

The P300 waveforms of average responses to standard stimuli do not have a well-defined single peak that can provide a unique amplitude and latency measure that can be incorporated into statistical analysis. Statistical analysis is therefore limited to the average responses to target stimuli where well-defined P300 waveforms make precise measurements possible. Figure 1 displays the grand average ERPs in response to target and standard stimuli at the six electrodes in Converters and Stables. Because the Oz channel was lost in some recordings, the statistical analysis is further limited to Fz, Cz, Pz, C3, and C4 electrode sites. We found that for all these electrode sites, the P300 amplitude was significantly smaller (*p* < 0.05) for the Converter group compared to the Stable group. The P300 latency was not significantly different (*p* > 0.05) between the two groups. The statistical results for each electrode were summarized in Table 3. We also explored the correlation between the P300 amplitudes and the psychological measures (CAPS, PHQ-9, and PCL-M) across subjects. No significant correlations were found (*p* > 0.05).

**Table 3:** **Baseline results from participants who remained PTSD-negative for one year after enrollment (*N* = 60) and those who converted to PTSD-positive (*N* = 5)**.

	Baseline scores individuals	Baseline scores individuals	Between-group separation	*P*_ERROR_
	PTSD-negative at 1 year stables	PTSD-positive at 1 year converters	*T*-test, two-tailed, unequal variance	Equal priors
	*N* = 60 (*N* = 59 for Fz)	*N* = 5	*p*	
Average Fz amplitude response to target stimulus (μV)	9.87 ± 4.25	5.98 ± 2.38	0.0157	0.3193
Average Cz amplitude response to target stimulus (μV)	15.48 ± 5.45	9.71 ± 2.65	0.0038	0.2937
Average Pz amplitude response to target stimulus (μV)	16.18 ± 5.27	11.13 ± 3.71	0.0338	0.3130
Average C3 amplitude response to target stimulus (μV)	14.52 ± 5.16	9.34 ± 2.54	0.0054	0.3032
Average C4 amplitude response to target stimulus (μV)	14.72 ± 5.23	9.07 ± 2.67	0.0046	0.2898
Average Fz latency response to target stimulus (ms)	356.2 ± 43.8	357.0 ± 57.2	0.9760	0.4963
Average Cz latency response to target stimulus (ms)	359.7 ± 39.0	357.3 ± 57.6	0.9235	0.4868
Average Pz latency response to target stimulus (ms)	360.5 ± 42.4	374.2 ± 58.6	0.6345	0.4377
Average C3 latency response to target stimulus (ms)	359.4 ± 37.3	352.3 ± 68.2	0.8291	0.4646
Average C4 latency response to target stimulus (ms)	355.2 ± 36.9	355.5 ± 60.3	0.9928	0.4987

## Diagnostic Validity

### Approximate Classification Error Rate

As summarized in Table 3, there was a statistically significant difference in the target amplitude between the participants who remained PTSD-negative throughout the study and those who became PTSD-positive. A statistically significant between-group separation does not, however, establish the efficacy of these measures as predictors. The most commonly applied quantitative measure of between-group separation is the *t*-test. As shown in Table 3, a naive calculation (a two-tailed *t*-test that assumes unequal variances) suggests a significant separation between the two participant groups. Two essential observations should be made. First, the asymptotic assumptions of the *t*-test cannot be meaningfully satisfied when *N*_C_ = 5. Second, a separation of means, which is what the *t*-test assesses, does not of itself ensure a successful classification even in those instances where the assumptions of the test are satisfied. An estimate of classification error rates can be made by again assuming normality of the two populations. The equations used are given in the Mathematical Appendices. This estimate often results in a substantial under estimate of the true error rate. This is particularly true when population numbers are small (58). The results shown in Table 3 show that application of this admittedly optimistic error rate estimate predicts that using target amplitude results in unacceptable classification error rates of *P*_ERROR_ = 0.29 to *P*_ERROR_ = 0.32, where it should be remembered that random assignment results in a 0.50 error if we assume that the two populations occur in equal proportions. This negative conclusion will be supported by the more reliable empirical determinations of classification error. It should be noted, however, that the error rates are different between the amplitudes and latencies, namely approximately 30% for the amplitudes and 50% for the latencies.

### ROC Curve

**Figure 2:**
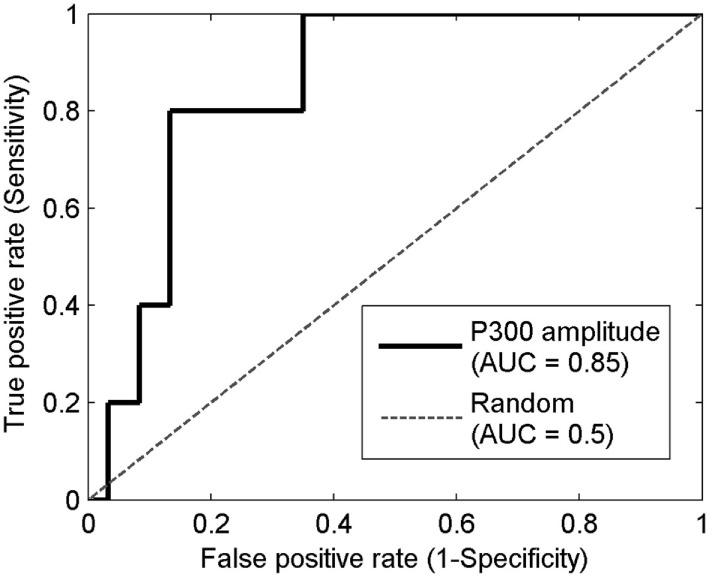
**The receiver operating characteristic (ROC) curve of the P300 amplitude at Cz**. Horizontal axis is the false positive rate (1-specificity) which equals the number of false positive divided by the sum of false positive and true negative. Vertical axis is the true positive rate (sensitivity) which equals the number of true positives divided by the sum of true positive and false negative. The solid line represents the ROC curve for using the P300 amplitude at Cz as the diagnostic test. The dashed line represents the ROC curve for a random test.

Prediction using prodromes can be treated as a diagnostic problem in which the disease-positive state corresponds to being a member of the group that becomes PTSD positive. Calculation of the ROC curve is a commonly employed method for characterizing a diagnostic classification. The first row of Table 4 shows the area under the curve (AUC), for the electrophysiological measures. The mathematical methods used to determine the AUC and its confidence intervals are given in the Mathematical Appendices. A value of AUC >0.5 indicates better than random assignment. The P300 amplitude at Cz showed the highest predictive power, with an AUC of 0.85 (confidence interval of [0.67, 0.94]). The ROC curve of the P300 amplitude at Cz is shown in Figure 2. While the values of the AUC are encouraging, the very large confidence intervals diminish confidence in the result.

**Table 4:** **Area under the receiver operating curve and measures of diagnostic efficacy computed using the smallest value of threshold giving the maximum value of the Youden index**.

Measure	Average Fz amplitude response to target stimulus	Average Cz amplitude response to target stimulus	Average Pz amplitude response to target stimulus	Average C3 amplitude response to target stimulus	Average C4 amplitude response to target stimulus
Area under the curve	0.7864 [0.5616, 0.8960]	0.8533 [0.6708, 0.9347]	0.7833 [0.4737, 0.9108]	0.8233 [0.6170, 0.9185]	0.8433 [0.5980, 0.9390]
Max Youden Index	0.5763	0.6667	0.5500	0.6000	0.7000
*T*_MAX_ (μV)	9.0140	10.4186	12.1866	12.4016	9.1688
Diagnostic accuracy	0.6094	0.8615	0.7538	0.6308	0.8923
Sensitivity	1.0000	0.8000	0.8000	1.0000	0.8000
Specificity	0.5763	0.8667	0.7500	0.6000	0.9000
Positive likelihood ratio	2.3600	6.0000	3.2000	2.5000	8.0000
Negative likelihood ratio	0.0000	0.2308	0.2667	0.0000	0.2222
Diagnostic odds ratio	Undefined	26.0000	12.0000	Undefined	36.0000

### Diagnostic Efficacy and Determination of the Diagnostic Cut Score

The results of a diagnostic calculation (and by implication for the present context the identification of a prodrome) can be expressed in the canonical four element diagnostic matrix: true positive, false positive, false negative, and true negative. There is no single fully satisfactory summary measure for characterizing the diagnostic matrix. Each has advantages and limitations. The limitations are particularly evident in studies like this one where disease prevalence is low. We will therefore examine six common measures of diagnostic efficacy: diagnostic accuracy, sensitivity, specificity, the positive likelihood ratio, the negative likelihood ratio, and the diagnostic odds ratio. Their definitions are given in the Mathematical Appendices.

The values of elements in the diagnostic matrix, and therefore measures of diagnostic efficacy like sensitivity and specificity, are critically dependent on the cut score used to assign individuals to the disease-positive and disease-negative groups. The choice of the cut value is therefore a central problem in the implementation of a diagnostic procedure. As outlined in the Mathematical Appendices, more than one candidate procedure has been proposed. In the calculations summarized in Table 4, the diagnostic threshold was determined by the value of threshold that gave the maximum value of *J*, the Youden index (59). The value of sensitivity, specificity, and other measures of diagnostic efficacy reported in Table 4 are the values obtained when the threshold was set to the smallest value of threshold giving the maximum *J*. Because the results of Table 3 indicate that target latencies cannot discriminate between-group means, the analysis is limited to target amplitudes.

### Leave-One-Out Cross Validation

The results presented in Table 4 are encouraging particularly in the cases of average Cz amplitude and average C4 amplitude which give sensitivity and specificity values in excess of 0.8. Measures of diagnostic efficacy obtained by examination of the ROC can be misleadingly optimistic if sample sizes are small. A fast, albeit imperfect, reality check can be implemented by a leave-one-out cross validation. In this calculation, one of the values is removed from the sample. A between-group classifier is constructed from the remaining data, and the omitted value is classified. It is then replaced. Another value is removed and classified. This procedure continues to exhaustion and the classification results are used to populate the diagnostic matrix (true positive, false positive, false negative, true negative). The measures of diagnostic efficacy introduced in the previous section are then calculated.

In order to implement a leave-one-out cross validation the choice of classifier must be addressed. In these calculations, a classifier based on Gaussian populations with prior probabilities was used. The mathematical structure of the classifier is given in the Mathematical Appendices. Two sets of prior probabilities were considered. In the first set of calculations, equal priors were used. In the second, it was supposed that the prior probability of delayed-onset PTSD was 0.25 which is the value derived from a review of the clinical literature (19, 20).

With both sets of prior probabilities, the sensitivity and specificity values are considerably less encouraging (Table 5). In the previous calculations, the sensitivity and specificity obtained at Cz are 0.80 and 0.87, respectively. In the leave-one-out calculation using equal priors, the corresponding values are 0.60 and 0.65. Similarly, the previous sensitivity and specificity results obtained at C4 were 0.80 and 0.90, respectively. The leave-one-out values with equal priors are 0.80 and 0.62. This divergence counsels interpretive caution when evaluating the results summarized in Table 3.

**Table 5:** **Classification based on average target amplitudes determined by a leave-one-out calculation**.

Measure	Average Fz amplitude response to target stimulus	Average Cz amplitude response to target stimulus	Average Pz amplitude response to target stimulus	Average C3 amplitude response to target stimulus	Average C4 amplitude response to target stimulus
**Prior probabilities, *P*_s_ = 0.5, *P*_c_ = 0.5**	
Number of true positives	4	3	4	3	4
Number of false positives	25	21	25	24	23
Number of false negatives	1	2	1	2	1
Number of true negatives	34	39	35	36	37
Diagnostic accuracy	0.5938	0.6462	0.6000	0.6000	0.6308
Sensitivity	0.8000	0.6000	0.8000	0.6000	0.8000
Specificity	0.5763	0.6500	0.5833	0.6000	0.6167
**Prior probabilities, *P*_s_ = 0.75 *P*_c_ = 0.25**	
Number of true positives	0	1	0	1	3
Number of false positives	6	7	3	7	8
Number of false negatives	5	4	5	4	2
Number of true negatives	53	53	57	53	52
Diagnostic accuracy	0.8281	0.8308	0.8769	0.8308	0.8462
Sensitivity	0.0000	0.2000	0.0000	0.2000	0.6000
Specificity	0.8983	0.8833	0.9500	0.8833	0.8667

### Populating the Diagnostic Matrix by Bootstrapping

A deficiency of the results presented in the previous section is immediately apparent on examining Table 5. The sensitivities and specificities are reported without confidence intervals. This deficiency can be addressed with a bootstrap calculation. The procedure is outlined in the Mathematical Appendices. Two thousand bootstrap samples were used to estimate the bootstrapped distribution. The results are shown in Table 6. The confidence intervals provide an essential clarification to the preceding results. The sample size precludes a dispositive response to the hypothesis that the amplitudes of average ERPs can serve as a predictor of delayed-onset PTSD.

**Table 6:** **Classification based on average target amplitudes determined by a bootstrap calculation**.

Measure	Average Fz amplitude response to target stimulus	Average Cz amplitude response to target stimulus	Average Pz amplitude response to target stimulus	Average C3 amplitude response to target stimulus	Average C4 amplitude response to target stimulus
**Prior probabilities *P*_s_ = 0.5, *P*_c_ = 0.5**	
Diagnostic accuracy	0.6288 [0.4348, 0.8261]	0.6719 [0.4500, 0.8621]	0.6087 [0.3333, 0.8462]	0.6431 [0.4500, 0.8261]	0.6954 [0.4286, 0.9091]
Sensitivity	0.6236 [0.0000, 1.0000]	0.5916 [0.0000, 1.0000]	0.6835 [0.0000, 1.0000]	0.5790 [0.0000, 1.0000]	0.6746 [0.0000, 1.0000]
Specificity	0.6325 [0.4118, 0.9048]	0.6802 [0.4211, 0.9444]	0.6068 [0.3158, 0.9444]	0.6513 [0.4211, 0.8846]	0.6996 [0.3913, 1.0000]
Positive likelihood ratio	1.6108 [0.4423, 3.2051]	1.8999 [0.5882, 4.6667]	1.6975 [0.3333, 3.8182]	1.5957 [0.4058, 3.3409]	2.6276 [0.7667, 7.0000]
Negative likelihood ratio	0.6720 [0.2174, 1.3889]	0.6592 [0.2121, 1.2857]	0.6695 [0.2069, 1.8254]	0.6992 [0.2114, 1.4457]	0.5572 [0.2100, 1.1613]
Diagnostic odds ratio	3.7930 [0.3176, 11.9231]	4.3037 [0.4667, 15.6154]	4.0970 [0.2000, 13.8889]	3.9384 [0.2870, 13.8889]	5.9901 [0.6863, 19.8000]
**Prior probabilities *P*_s_ = 0.75, *P*_c_ = 0.25**	
Diagnostic accuracy	0.8282 [0.6364, 0.9583]	0.8159 [0.6400, 0.9545]	0.8647 [0.6818, 0.9615]	0.8023 [0.6190, 0.9565]	0.8562 [0.7143, 0.9583]
Sensitivity	0.1533 [0.0000, 1.0000]	0.3104 [0.0000, 1.0000]	0.1481 [0.0000, 1.0000]	0.2175 [0.0000, 1.0000]	0.3513 [0.0000, 1.0000]
Specificity	0.8887 [0.6667, 1.0000]	0.8622 [0.6667, 1.0000]	0.9269 [0.7200, 1.0000]	0.8542 [0.6522, 1.0000]	0.9030 [0.7273, 1.0000]
Positive likelihood ratio	4.0682 [0.5778, 12.5000]	3.3854 [0.6786, 12.0000]	5.5863 [0.7407, 15.7500]	3.0264 [0.4902, 12.0000]	4.9856 [0.9286, 16.5000]
Negative likelihood ratio	0.8286 [0.2949, 1.1667]	0.7378 [0.2660, 1.1111]	0.7963 [0.2805, 1.0811]	0.8170 [0.2838, 1.2069]	0.6722 [0.2000, 1.0135]
Diagnostic odds ratio	6.1093 [0.4921, 19.8000]	6.6509 [0.6104, 29.4000]	8.6072 [0.6863, 39.0000]	5.1757 [0.4026, 23.4000]	10.2212 [0.9184, 43.0000]

The confidence intervals reported for sensitivity values, [0,1] in all cases, are particularly telling. The definition of sensitivity is

$$\text{Sensitivity}\;=\;\text{True Positive Ratio}\;=\;\frac{N_{\text{TP}}}{N_{\text{TP}}+N_{\text{FN}}}$$

where *N*_TP_ is the number of true positives and *N*_FN_ is the number of false negatives. There are only five elements in the Converter set, and two of these elements are used to build the classifier. Therefore, *N*_TP_ is frequently zero, giving Sensitivity = 0. Similarly, if in other cases *N*_TP_ ≠ 0 and *N*_FN_ = 0 giving Sensitivity = 1 as another frequent value. This results in a bootstrapped confidence interval of [0,1].

## Discussion

In this analysis, the identification of individuals who will present delayed-onset PTSD is treated as a diagnostic process where the diagnostic groups are Converters (those who present delayed-onset PTSD) and Stables (those who do not). Sensitivity values based on average target stimulus amplitude range from 0.58 to 0.68. Specificity values range from 0.61 to 0.70, suggesting that event-related potentials may be helpful in identifying at-risk individuals.

The results in this study can only be considered preliminary due to the small sample size of Converters. The limitations of the sample size are indicated by the calculations presented in Table 6. Suppose the objective is to know sensitivity to an accuracy of ±0.1 with 95% confidence. A calculation given in the Mathematical Appendices indicates that *N* ≥ 185 is required, where it must be emphasized that this *N* is the number of Converters. If Converters are 10% of the population, then the projected requirement is for 1,850 participants in the study. The implications of this simple calculation extend beyond the study of PTSD and generalize to all of neuropsychiatry where conversion rates even in enriched populations are low. Large participant numbers will be required. Additionally, by definition, the search for prodromes requires a longitudinal study extended, perhaps, over a period of years. The challenges of supporting and implementing very large longitudinal studies are formidable.

Further limitations should be acknowledged. Electrophysiological abnormalities associated with neuropsychiatric disorders are non-specific. For example, in addition to PTSD, alterations in EEG synchronization have been observed in AD/HD, alcohol abuse, alexithymia, autism, bipolar disorder, dementia, depression, migraine, multiple sclerosis, Parkinson’s disease, TBI, schizophrenia, and other psychotic disorders (60). The potential loss of electrophysiological specificity is particularly likely in a military population where PTSD is often associated with TBI and is comorbid with depression and substance abuse. Additionally, medications can alter event-related potentials and will complicate diagnosis based on ERPs.

Statistical identification of individuals who will present with PTSD might, however, be improved by two extensions to the present analysis. First, the analysis of ERPs reported here was limited to calculation of average ERPs. More recently, developed methods of analysis, for example, information dynamics (61) and network analysis of brain electrical activity (62) might improve results. Second, specificity and sensitivity may be improved by combining electrophysiological measures with other biomarkers and clinical information. Incorporating scores from psychological questionnaires with electrophysiological results in a multivariate discrimination would be an obvious possibility. The psychological measures including CAPS, PHQ-9, and PCL-M scores showed significant difference between Stables and Converters at the baseline assessment, but none of the scores significantly correlated with the P300 amplitude. The discordance between neural responses and self-reported symptoms may be partially a consequence of psychological defensive denial (63, 64). Some SMs recruited in this study may deny the presence of their PTSD symptoms due to military training or concerns that this may jeopardize their job, promotion, and self-image. This defensive denial may be softened after a prolonged period. Consistent with this possibility, a review by Andrews et al. (19) reported that most delayed-onset PTSD cases occurred in military samples rather than in civilian samples. If this is the case, objective biomarkers would be fundamentally more favorable than self-report psychological measures in identifying SMs at risk of PTSD.

While additional forms of electrophysiological analysis in combination with other classes of data may improve the likelihood of success, this will not eliminate the previously documented requirement for large sample sizes in a longitudinal study. Such detection would be critical to the military because early intervention to prevent PTSD has revealed a critical window for fear activation and extinction of conditioned responses related to traumatic memories (65).

## Ethics Statement

This research protocol was approved by the Institutional Review Board of the Uniformed Services University and by the Institutional Review Board of the Walter Reed National Military Medical Center. All participants gave written informed consent in accordance with the Declaration of Helsinki.

## Author Contributions

CW performed the analysis of the event-related potentials and the preliminary statistical analysis. MC screened participants for eligibility and conducted the psychological assessments. PR performed the literature search, statistical analysis, and wrote the final drafts of the paper. DD participated in developing and implementing the statistical analysis plan. KB and DN obtained the electrophysiological data. CC designed and built the ERP acquisition system. MR participated in the design of the investigation. DK lead the research effort and participated in acquisition of the electrophysiological data.

## Conflict of Interest Statement

The authors declare that the research was conducted in the absence of any commercial or financial relationships that could be construed as a potential conflict of interest.

## Mathematical Appendices

### Estimating Classification Error (Contents of Table 3)

For the case of a single discriminating variable, the Group A–Group B between-group Mahalanobis distance is

$$\begin{array}{ll} D_{\text{A,B}}^{2} & =\frac{{\left({\hat{\mu }}_{\text{A}}-{\hat{\mu }}_{\text{B}}\right)}^{2}}{{\hat{\sigma }}_{\text{AB}}^{2}},\\ {\hat{\sigma }}_{\text{AB}}^{2} & =\frac{\left(N_{\text{A}}-1\right){\hat{\sigma }}_{\text{A}}^{2}+\left(N_{\text{B}}-1\right){\hat{\sigma }}_{\text{B}}^{2}}{N_{\text{A}}+N_{\text{B}}-2}, \end{array}$$

${\hat{\mu }}_{\text{A}}$ is the Group A sample mean, and ${\hat{\sigma }}_{\text{A}}$ is the Group A sample SD. ${\hat{\mu }}_{\text{B}}$ and ${\hat{\sigma }}_{\text{B}}$ are defined analogously. *P*_ERROR_ (*G*_A_, *G*_B_) is the error rate for the optimal classifier under the assumption of normality for the two populations and provides an estimate classification error when only means and SDs are known. It can give a serious underestimate of true classification error. This is especially true if group population numbers are low or the assumption of normality is violated. When full data sets are available, an empirical calculation of error rate is preferred *via* either cross-validation or bootstrapping. Let ρ_A_ and ρ_B_ be prior probabilities of Group A and Group B membership. *P*_ERROR_ (*G*_A_, *G*_B_) is given by

$$\begin{array}{ll} P_{\text{ERROR}}\left(G_{\text{A}},G_{\text{B}}\right) & =\rho_{\text{A}}\Phi \;\left\{\;\;-\frac{1}{2}\sqrt{D_{\text{A,B}}^{2}}+\frac{1}{\sqrt{D_{\text{A,B}}^{2}}}\log_{e}\left(\frac{\rho_{\text{B}}}{\rho_{\text{A}}}\right)\;\;\right\}\\ & \;\;+\rho_{\text{B}}\Phi \;\left\{\;\;-\frac{1}{2}\sqrt{D_{\text{A,B}}^{2}}-\frac{1}{\sqrt{D_{\text{A,B}}^{2}}}\log_{e}\left(\frac{\rho_{\text{B}}}{\rho_{\text{A}}}\right)\;\;\right\} \end{array}$$

where Φ(*x*) is the cumulative distribution function for a standard normal random variable (75). For the case of equal priors, the expression reduces to

$$P_{\text{ERROR}}\left(G_{\text{A}},G_{\text{B}}\right)=1-\Phi \;\left(\frac{\sqrt{D_{\text{A,B}}^{2}}}{2}\right)=\Phi \;\left(\frac{-\sqrt{D_{\text{A,B}}^{2}}}{2}\right)\;.$$

### Receiver Operating Characteristic Curve (Contents of Table 4)

The area under an empirical receiver operating characteristic curve is equal to the Mann–Whitney *U* statistic [Ref. (74), p. 65 following from a proof on p. 27] and thus the Mann–Whitney *U* statistic provides an estimator for the population level AUC. Random assignment results in AUC = 0.5. The following notation is introduced:

*N_S_* number of longitudinally stable participants
*N_C_* number of converter participants
*S_i_* observed value for the *i*-th stable participant
*C_j_* observed value for the *j*-th converter participant.

$$\hat{\text{AUC}}=\frac{1}{N_{C}N_{S}}\sum_{i=1}^{N_{S}}\sum_{j=1}^{N_{C}}\left\{I\left(C_{j}<S_{i}\right)+\frac{1}{2}I\left(C_{j}=S_{i}\right)\right\}$$

where *I*(*Z*) = 1 if argument *Z* is true. It is important to note that “less than” used in this application, contra textbooks where “greater than” appears, because in this analysis a participant is classed as positive if the observed value is less than the threshold value.

There are several estimates of the variance of the AUC [listed on p. 67 of Ref. (74)]. We use here the expression in Hanley and McNeil (71).

$$\begin{array}{ll} s^{2}\left(\hat{\text{AUC}}\right) & =\frac{1}{N_{S}N_{C}}\left\{\hat{\text{AUC}}\;\left(1-\hat{\text{AUC}}\right)+\left(N_{C}-1\right)\left(Q_{1}-{\hat{\text{AUC}}}^{\text{2}}\right)\right.\\ & \;\;\;\left.+\left(N_{S}-1\right)\left(Q_{2}-{\hat{\text{AUC}}}^{\text{2}}\right)\right\} \end{array}$$

As in the equation for AUC, the definition of *Q*_1_ and *Q*_2_ uses “less than” rather than “greater than” because a participant is classed as a positive if the measure value is below threshold rather than greater than threshold. *Q*_1_ is the proportion of all possible triples composed of two sampled members from the Converter group and one from the Stable group where the two Converter scores are less than the Stable score

$$Q_{1}=\frac{1}{N_{S}N_{C}N_{C}}\sum_{i=1}^{N_{S}}\sum_{j=1}^{N_{C}}\sum_{k=1}^{N_{C}}I\left(C_{j}<S_{i}\right)I\left(C_{k}<S_{i}\right)$$

*Q*_2_ is the proportion of all possible triples composed on one member from the Converter group and two members from the Stable group where the Converter score is less than both scores from the Stable group.

$$Q_{2}=\frac{1}{N_{C}N_{S}N_{S}}\sum_{i=1}^{N_{C}}\sum_{j=1}^{N_{S}}\sum_{k=1}^{N_{S}}I\left(C_{i}<S_{j}\right)I\left(C_{i}<S_{k}\right)$$

An expression for confidence intervals has been constructed by (80), where with confidence 1 − α, the true AUC lies in the interval given by

1−(1−AUC^)exp±z1−α∕2s2(AUC^)∕(1−AUC^),

where *z*_1−α/2_ is the 1 − α/2 quantile of a standard normal random variable. Under this transformation/inverse transformation, the upper and lower confidence intervals are always in the interval [0,1].

An analysis of the ROC can be used to determine the optimal cutoff value for a continuous, dichotomous diagnostic test. Glas et al. (70) have endorsed the diagnostic odds ratio as a single indicator of test performance and proposed using its maximum to determine the cutoff value. Pepe et al. (76) have argued against this practice and have provided examples that identify limitations of the odds ratio. A fundamental limitation is immediately apparent on examining the equation below for the ratio. It is undefined if the number of false positive or the number of false negatives is 0. Böhning et al. (66) have continued the analysis and recommend using the maximum value of the Youden index (59) as an alternative indicator of the best cutoff value. The Youden index, also called the Youden *J* statistic is

J=Sensitivity+Specificity−1.

It is reported as a function of threshold, and the recommended value of threshold is the lowest threshold value giving the maximum of *J*.

### Measures of Diagnostic Efficacy (Contents of Table 4)

Dichotomous diagnosis (two possible outcomes, disease positive and disease negative), using a single continuous variable is considered here. The diagnostic utility of the measure and classifier combination is investigated by first populating the diagnostic matrix and then computing standard measures of diagnostic efficacy. Six measures are considered here diagnostic accuracy, sensitivity, specificity, positive likelihood ratio, negative likelihood ratio, and the diagnostic odds ratio, where it should be recognized that no single measure of diagnostic effectiveness provides a complete assessment of a measure’s ability to classify participants (76). Additional measures are presented in Pepe (76) and in Portney and Watkins (78).

**Table:**

	Disease present	Disease absent
Test positive	True positive	False positive
	*N*_TP_	*N*_FP_
Test negative	False negative	True negative
	*N*_FN_	*N*_TN_

$$\text{Diagnostic}\;\text{Accuracy}=\left(N_{\text{TP}}+N_{\text{TN}}\right)∕\left(N_{\text{TP}}+N_{\text{FP}}+N_{\text{FN}}+N_{\text{TN}}\right)$$

$$\text{Sensitivity}=\text{True Positive Fraction}=\frac{N_{\text{TP}}}{N_{\text{TP}}+N_{\text{FN}}}$$

$$\text{Specificity}=\text{True Negative Fraction}=\frac{N_{\text{TN}}}{N_{\text{TN}}+N_{\text{FP}}}$$

$$\begin{array}{ll} \text{Positive Likelihood Ratio} & =\text{LR}+=\frac{\text{True Positive Fraction}}{\text{False Positive Fraction}}\\ & =\left(\frac{N_{\text{TP}}}{N_{\text{TP}}+N_{\text{FN}}}\right)/\left(\frac{N_{\text{FP}}}{N_{\text{FP}}+N_{\text{TN}}}\right)\\ & =\frac{\text{Sensitivity}}{1-\text{Specificity}} \end{array}$$

$$\begin{array}{ll} \text{Negative Likelihood Ratio} & =\text{LR}-=\frac{\text{False Negative Fraction}}{\text{True Negative Fraction}}\\ & =\left(\frac{N_{\text{FN}}}{N_{\text{FN}}+N_{\text{TP}}}\right)/\left(\frac{N_{\text{TN}}}{N_{\text{TN}}+N_{\text{FP}}}\right)\\ & =\frac{1-\text{Sensitivity}}{\text{Specificity}} \end{array}$$

$$\begin{array}{ll} \text{Diagnostic Odds Ratio} & =\text{DOR}=\frac{\text{LR}+}{\text{LR}-}=\left(\frac{N_{\text{TP}}\cdot N_{\text{TN}}}{N_{\text{FP}}\cdot N_{\text{FN}}}\right)\\ & =\left(\frac{\text{Sensitivity}}{1-\text{Sensitivity}}\right)\left(\frac{\text{Specificity}}{1-\text{Specificity}}\right) \end{array}$$

### Classification Based on Gaussian Likelihood and Bayesian Priors (Contents of Table 5)

The classifier is constructed from a single continuous variable, in this case the amplitude of the average response to the target stimulus. ${\left\{S_{j}\right\}}_{j=1}^{N_{S}}$ is the set of values obtained from clinically stable participants. ${\hat{\mu }}_{S}$ is the sample mean and ${\hat{\sigma }}_{S}$ is the corresponding SD. ${\left\{C_{j}\right\}}_{j=1}^{N_{c}}$ is the set of values obtained from participants who became PTSD-positive and has mean ${\hat{\mu }}_{C}$ and SD ${\hat{\sigma }}_{C}$. Let *x* denote the value of the measure obtained from the individual who is to be classified. The group specific density function for clinically stable participants is

fS(x)=1(2πσ^S2)1∕2exp−12(x−μ^S)2∕σ^S2

*f_C_*(*x*) is defined analogously. The posterior probabilities of group membership are

$$P\left(G=S|x\right)\frac{\rho_{S}{\;f}_{S}\left(x\right)}{\rho_{S}{\;f}_{S}\left(x\right)+\rho_{C}{\;f}_{C}\left(x\right)}$$

and

$$P\left(G=C|x\right)\frac{\rho_{C}{\;f}_{C}\left(x\right)}{\rho_{S}{\;f}_{S}\left(x\right)+\rho_{C}{\;f}_{C}\left(x\right)}$$

where ρ*_S_* and ρ*_C_* are the prior probabilities of membership in the healthy or disease-positive groups. The participant presenting measure equal to *x* is classified into the group with the higher posterior probability.

### Populating the Diagnostic Matrix with a Bootstrap Estimator (Contents of Table 6)

A bootstrap (67) can be used to determine the value of the diagnostic metrics, and the corresponding confidence intervals. The procedure used here is similar to the bootstrap cross validation scheme for small sample sizes implemented by Jiang et al. (73). A procedure for finding the best estimate of Sensitivity from the available data is described here. The procedure immediately generalizes to other measures of diagnostic efficacy.

As before, this presentation describes a dichotomous classification using a single continuous variable between two groups, clinically stable participants and participants presenting delayed onset PTSD. ${\left\{S_{j}\right\}}_{j=1}^{N_{S}}$ is the set of values of this measure obtained from clinically stable participants. There are *N_S_* elements. ${\left\{C_{j}\right\}}_{j=1}^{N_{c}}$ is the set of values obtained from participants who convert to the PTSD-positive state. There are *N_C_* values. A single iteration of the bootstrap proceeds as follows:

1. *N_C_* + *N_S_* elements are drawn randomly with replacement from the combined set ${\left\{S_{j}\right\}}_{j=1}^{N_{S}}\cup {\left\{C_{j}\right\}}_{j=1}^{N_{C}}$. This set of randomly drawn elements is denoted by ${\left\{B_{j}\right\}}_{j=1}^{N_{S}+N_{C}}$, the bootstrap sample. Typically, the bootstrap sample will contain repeated values. It is possible that the bootstrap sample does not contain an element from either set ${\left\{S_{j}\right\}}_{j=1}^{N_{S}}$ or set ${\left\{C_{j}\right\}}_{j=1}^{N_{c}}$. If this occurs, this iteration of the bootstrap is ignored. Additionally, depending on the classifier used, a minimum number of distinct values from ${\left\{S_{j}\right\}}_{j=1}^{N_{S}}$ and ${\left\{C_{j}\right\}}_{j=1}^{N_{c}}$ will be required to construct the classifier. For example, the classifier based on Gaussian population densities will require at least two distinct elements from each set. If this minimum requirement is not satisfied this iteration of the bootstrap is ignored and the process returns to the beginning of Step 1. Also, if there is not at least one element of ${\left\{S_{j}\right\}}_{j=1}^{N_{S}}$ and one element of ${\left\{C_{j}\right\}}_{j=1}^{N_{c}}$ in the set of elements that will be classified, the randomization is rejected and the process returns to the beginning of Step 1.
2. The class membership of each element of ${\left\{B_{j}\right\}}_{j=1}^{N_{S}+N_{C}}$ is known. Use ${\left\{B_{j}\right\}}_{j=1}^{N_{S}+N_{C}}$ to construct a classifier.
3. Use this classifier to classify all members of the combined set ${\left\{S_{j}\right\}}_{j=1}^{N_{S}}\cup {\left\{C_{j}\right\}}_{j=1}^{N_{C}}$ not in ${\left\{B_{j}\right\}}_{j=1}^{N_{S}+N_{C}}$, namely $\left\{{\left\{S_{j}\right\}}_{j=1}^{N_{S}}\cup {\left\{C_{j}\right\}}_{j=1}^{N_{C}}\right\}\setminus {\left\{B_{j}\right\}}_{j=1}^{N_{S}+N_{C}}$. The results of this classification are used to calculate *N*_TP_, *N*_FP_, *N*_FP_, *N*_TN_ specific to this bootstrap sample. Though in the general case, it is possible, but unlikely, that $\left\{{\left\{S_{j}\right\}}_{j=1}^{N_{S}}\cup {\left\{C_{j}\right\}}_{j=1}^{N_{C}}\right\}\setminus {\left\{B_{j}\right\}}_{j=1}^{N_{S}+N_{C}}$ is the null set, this will not occur in the present application because of the constraints on the randomization put in place in Step 1.
4. Sensitivity and other measures of diagnostic efficacy for this iteration of the bootstrap are then calculated using standard formulas.

This process is repeated until *N_B_* values of Sensitivity are obtained. This may require more than *N_B_* iterations of the bootstrap if the requirements of the random sample outlined in Step 1 are not met.

The average value of sensitivity, computed from the *N_B_* successful iterations of the bootstrap is the best available estimate from ${\left\{S_{j}\right\}}_{j=1}^{N_{S}}$ and ${\left\{C_{j}\right\}}_{j=1}^{N_{c}}$. The confidence interval of sensitivity can be determined from the distribution of the *N_B_* values of sensitivity. For example, suppose that sensitivities are calculated from 2,000 bootstrap samples and suppose that the 95% confidence interval is to be determined. Rank order the values of sensitivity. The lower bound of the confidence interval is the 50th element, and the upper bound is element 1950th.

This leaves the specification of *N_B_* as an open question. This is not a question that has a single answer (68, 69). The required number of iterations will depend on what is being estimated and the properties of the underlying distribution. A convention in the community regards *N_B_* = 1,000 as a lower bound. As an operational suggestion the estimate of sensitivity, for *N_B_* = 1,000 and *N_B_* = 2,000 can be compared. *N_B_* should be large enough to give a stable value of sensitivity. *N_B_* = 2,000 was used in these calculations.

This is a constrained randomization. At least two distinct elements of each class (Stables and Converters) must be in the set used to construct the classifier $\left(\text{the Training Set},{\left\{B_{j}\right\}}_{j=1}^{N_{S}+N_{C}}\right)$. At least one element of each class must be in the set that is classified $\left(\text{the Testing Set}={\left\{S_{j}\right\}}_{j=1}^{N_{S}}\cup {\left\{C_{j}\right\}}_{j=1}^{N_{C}}\setminus {\left\{B_{j}\right\}}_{j=1}^{N_{S}+N_{C}}\right)$. Because at least one element of ${\left\{C_{j}\right\}}_{j=1}^{N_{c}}$ is classified, there will be at least one true positive (a converter assigned into the converter group) or one false negative (a converter classified into the stable group). Sensitivity may be 0 (*N*_TP_ = 0), but it will not be singular because *N*_TP_ + *N*_FN_ ≠ 0. Because there are only five elements of ${\left\{C_{j}\right\}}_{j=1}^{N_{c}}$ and two are used to build the classifier, *N*_TP_ is, however, frequently 0, and Sensitivity = 0 is therefore a frequent result from an iteration of the bootstrap. Additionally in many other cases, *N*_TP_ ≠ 0, but *N*_FN_ = 0 giving Sensitivity = 1. This explains the confidence interval of [0,1].

Because at least one element of ${\left\{S_{j}\right\}}_{j=1}^{N_{S}}$ will be classified, there is at least one true negative or one false positive. Therefore, since *N*_TN_ + *N*_FP_ ≠ 0, specificity will be defined at each iteration of the bootstrap. In contrast with Sensitivity, because ${\left\{S_{j}\right\}}_{j=1}^{N_{S}}$ is a much larger set, Specificity typically shows values different from 0 to 1.

The positive likelihood ratio is undefined if Specificity is equal to 1. As noted in the preceding paragraph this is unlikely, but it is possible. The negative likelihood ratio is undefined if Specificity is equal to 1. This frequently occurs with these data. The diagnostic odds ratio is undefined if either Specificity or Sensitivity is equal to 1. Glas et al. [(70), p. 1131] suggests adding 0.5 to all four elements of the diagnostic matrix in those applications where undefined values of the diagnostic ratios are likely to occur. This was done in these calculations.

### Sample Size Requirements for Measures of Diagnostic Efficacy

Sample size requirements for sensitivity and specificity assessments can be computed using an argument based on Hoeffding’s inequality [(72, 79), p. 65]. If α is the significance level for a confidence interval of length 2Δ, we require

$$\alpha \leq 2e^{-2N\Delta^{2}}$$

giving

$$N\geq \frac{-\ln\left(\alpha ∕2\right)}{2\Delta^{2}}$$

The sample size required for a ±0.1 sensitivity estimate with 95% (α = 0.05) confidence is seen to be *N* ≥ 185. It should be stressed that this is an estimate of sensitivity. *N* in this equation is the number of individuals in the sample who are disease positive. If the prevalence of the disorder in the enrollment population is 10%, then an enrollment ≥1,850 is required.
